# Molecular Characterization and Clinical Impact of *TMPRSS2-ERG* Rearrangement on Prostate Cancer: Comparison between FISH and RT-PCR

**DOI:** 10.1155/2013/465179

**Published:** 2013-05-28

**Authors:** A. Fernández-Serra, L. Rubio, A. Calatrava, J. Rubio-Briones, R. Salgado, R. Gil-Benso, B. Espinet, Z. García-Casado, J. A. López-Guerrero

**Affiliations:** ^1^Laboratory of Molecular Biology, Fundación Instituto Valenciano de Oncología, C/Professor Beltrán Báguena 8, 46009 Valencia, Spain; ^2^Department of Pathology, Fundación Instituto Valenciano de Oncología, C/Professor Beltrán Báguena 8, 46009 Valencia, Spain; ^3^Department of Urology, Fundación Instituto Valenciano de Oncología, C/Professor Beltrán Báguena 8, 46009 Valencia, Spain; ^4^Laboratori de Citogenètica Molecular, Servei de Patologia, GRETNHE, Programa de Recerca en Càncer, IMIM, Institut de Recerca del Hospital del Mar, Parc de Salut Mar, 08003 Barcelona, Spain; ^5^Department of Pathology, Universitat de Valencia Estudi General, 46010 Valencia, Spain

## Abstract

Prostate cancer (PCa) is a very heterogeneous disease, and there are constraints in its current diagnosis. Serum PSA levels, digital rectal examination (DRE), and histopathologic analysis often drive to overdiagnosis and overtreatment. Since 2005, the presence of the genetic rearrangement between transmembrane-serine protease gene (*TMPRSS2*) and the erythroblast transformation-specific (*ETS*) member *ERG* (v-ets erythroblastosis virus E26 oncogene homolog avian) has been demonstrated in almost half of PCa cases. Both FISH and RT-PCR are useful tools for detecting these rearrangements, but very few comparatives between both techniques have been published. In this study, we included FFPE tumors from 294 PCa patients treated with radical prostatectomy with more than 5 years of followup. We constructed a total of 20 tissue microarrays in order to perform break-apart and tricolor probe FISH approaches that were compared with RT-PCR, showing a concordance of 80.6% (P < 0.001). The presence of *TMPRSS2-ERG* rearrangement was observed in 56.6% of cases. No association between *TMPRSS2-ERG* status and clinicopathological parameters nor biochemical progression and clinical progression free survival was found. In conclusion, this study demonstrates that both FISH and RT-PCR are useful tools in the assessment of the *TMPRSS2-ERG* fusion gene status in PCa patients and that this genetic feature per se lacks prognostic value.

## 1. Introduction

Prostate cancer (PCa) is a heterogeneous disease, which ranges from indolent to lethal behaviour [1]. The diagnosis may be clinically suspect based on an elevated serum prostate specific antigen (PSA) and/or abnormal digital rectal examination (DRE), the definitive diagnosis established by histopathologic examination of needle biopsy tissue. However, both PSA and DRE often lead to both overdiagnosis and overtreatment presenting limitations when differentiating between indolent and aggressive PCa [2]. In addition, biopsy is also far from being optimal because it has demonstrated a lack of sensitivity and high risk of morbidity for patients [3]. Hence, in this context, there is an increasing demand of specific biomarkers for PCa diagnosis that also provides information regarding the prognosis of the disease.

In 2005 a novel set of fusion genes were described in nearly half of the PCa cases [4] involving the 5′-untranslated region of *TMPRSS2* (21q22) and the codifying region of some transcription factors such as *ERG* (21q22), *ETV1* (7p21), and *ETV4* (17q21) among others [5]. To date, there are 10 different genes involved in these genetic fusions [6]. However, *TMPRSS2-ERG *is the most prevalent, and more than 20 variants of the *TMPRSS2-ERG* fusion transcripts have been described [6, 7], and the exon 1 of *TMPRSS2* with the exon 4 or 5 of *ERG* (T1E4 or T1E5, resp.) are the two most frequently involved variants [8]. The main mechanisms by which the *TMPRSS2-ERG* fusion genes are produced are interstitial deletion and balanced translocation [9]. Because of their specificity, detection of these fusion genes could be a valuable ancillary diagnostic tool in the early detection of PCa [10]. In fact, these rearranged genes can be detected either by fluorescence in situ hybridization (FISH), reverse transcription polymerase chain reaction (RT-PCR) techniques [7, 11–14], or branched DNA (bDNA) analysis that is a very sensitive approach [15].

FISH is considered the gold standard in the detection of fusion rearrangements; the break-apart strategy is the main approach used for this propose [12]. Yoshimoto et al. developed a three-colour assay able to distinguish between the two main mechanisms of gene rearrangement for *TMPRSS2-ERG*, the interstitial deletion, or the reciprocal translocation [16]. In this sense, a commercial FISH assay comprising three-color (tricolor) probes has been developed that are able to discriminate between the putative fusion gene partners and the different gene rearrangement mechanisms.

The aim of this study is to compare FISH and RT-PCR techniques in the assessment of the *TMPRSS2-ERG* fusion gene in a series of 294 cases of PCa and to establish the prognostic usefulness of a commercial FISH tricolor deletion probe.

## 2. Material and Methods

### 2.1. Case Selection

Formalin fixed and paraffin-embedded (FFPE) blocks corresponding to PCa patients were retrieved from the archives of the Biobank of the *Fundación Instituto Valenciano de Oncología* according to the following criteria: radical prostatectomy specimens and no history of previous treatment for PCa (including androgen deprivation therapy or chemotherapy prior to surgery). We obtained 294 PCa specimens that met these criteria during the period between 1996 and 2008. All patients gave written informed consent, and the study was approved by the Ethics Committee of our institution (reference no. 2006-07). The clinical data were reviewed from the clinical records and stored in a PCa-specific database. The main characteristics and patient demographics are shown in Table 1. In addition, we also analyzed 20 samples of normal prostate tissue as controls obtained from radical cystectomies neither of which was benign prostatic hyperplasia or PCa. Haematoxylin and eosin (H&E) stained slides were reviewed and Gleason pattern was assigned.

### 2.2. TMAs Construction

We constructed 19 tissue microarrays (TMAs) comprising 294 tumor samples and another TMA with 20 samples of normal prostatic tissue used as control, containing three representative nonnecrotic cores of each case (1 mm in diameter). TMAs were constructed using a manual tissue arrayer (Beecher instruments, Silver Spring, MD).

### 2.3. Fluorescence In Situ Hybridization (FISH)

*TMPRSS2-ERG* fusion status was determined by using both a break-apart assay and a triple-labelled colour commercial probe KBI-10726 (Poseidon, Kreatech Diagnostics, The Netherlands) flanking both *TMPRSS2* and *ERG*. This probe is designed to detect the deletion between *TMPRSS2* and *ERG* at 21q22 region but also translocations involving this region with other genes such as *ETV1* or *ETV4*. 

For the break-apart assay we used two noncommercial flanking probes (red (R) and green (G)) to *ERG* to detect rearrangements affecting this gene [5]. BAC clones covering both distal and proximal regions of *ERG* on chromosomes 21q22, CTD-2341018, and CTD-219A22, respectively, were obtained from Children's Hospital Oakland Research Institute (Oakland, CA, USA). The selection of these BAC clones was performed using the genome browser from *Centre de Regulació Genòmica*, Barcelona, Spain (http://davinci.crg.es/). BAC DNA isolation was performed according to Qiagen Plasmid MIDI kit protocol (Qiagen Inc., Valencia, CA) and was labelled using a nick translation kit (AbbottMolecular, Abbott Park, IL). CTD-219A22 and CTD-2341018 BACs were labelled with the Spectrum Red-dUTP (AbbottMolecular) and Spectrum Green-dUTP (AbbottMolecular), respectively. The cytogenetic localization of all BACs was verified by hybridization to normal metaphase spreads (G-banding with inverted DAPI).

The FISH assay was carried out on 3 *μ*m thick FFPE tissue sections. After deparaffinization, tissue sections were treated with a commercial FFPE tissue section kit (MAD-FISH-PKII, Master Diagnostica, Granada, Spain) following the manufacturer's instructions. Briefly, the slides were immersed in thiocyanate solution at 80°C for 30 minutes and then treated with a protease solution for 10 minutes at 37°C. Afterwards, the tissue sections were pretreated and probes were denatured at 80°C for 5 minutes and hybridized overnight at 37°C using the hybridization System HyCrome (Euroclone S.pA., MI, Italy). Posthybridization washes were performed using a posthybridization solution (MAD-FISH-PKII, Master Diagnostica, Granada, Spain) for 2 minutes at 72°C and then, the slides were counterstained with 7 *μ*L of 4, 6-diamino-2-phenylindole (DAPI II, Vysis, Downers Grove, IL). Results were visualized using a ZEISS, Imager.Z1 fluorescent microscope with the AxioCam HRc camera the AxioVision 4 software (Carl ZEISS MicroImaging GmbH, Germany).

### 2.4. Criteria for the FISH Interpretation

**Figure 1:**
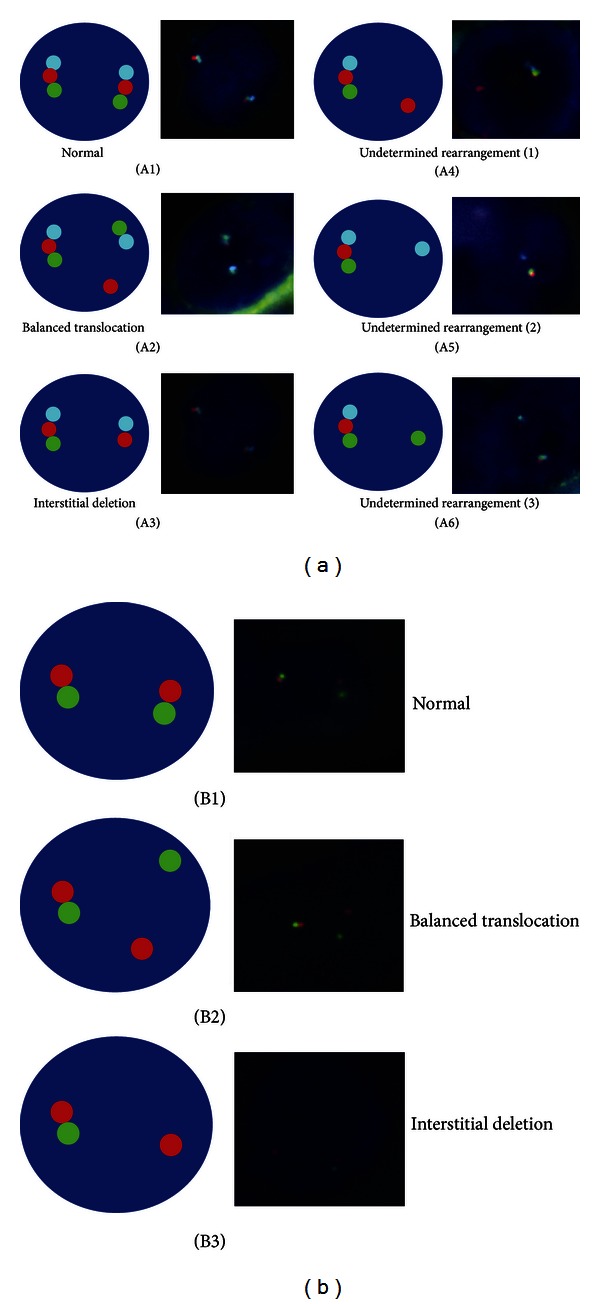
(a) Patterns obtained in the tricolor probe FISH analysis. Three fused signals indicate two normal alleles (A1); a balanced translocation pattern is considered with a fused (RGB) signal and an adjacent BG with a separated R signals (A2); interstitial deletion is indicated by a RGB signal and a B and R adjacent signals (A3); one RGB signal plus only one R, B, or G signal (A4, A5, A6, resp.) corresponds to undetermined rearrangements. (b) Patterns of the dual color break-apart assay: normal pattern with two RG signals (B1); balanced translocation is indicated by a RG signal plus a G and R separated signals (B2); one RG signal plus a R signal indicates interstitial deletion (B3).

The main mechanisms of the *TMPRSS2-ERG* rearrangement are interstitial deletion or translocation [17]. In our break-apart strategy, two yellow (Y) or R and G adjacent signals indicate a normal pattern, with no rearrangement in any of the two alleles. Whereas one Y and one R or G signal alone or one Y signal plus one R and one G separated signals indicate interstitial deletion and translocation, respectively. Thirty-eight cases of PCa were evaluated using break-apart probe in order to compare this approach with tricolor probes (Figure 1).

The Poseidon *TMPRSS2-ERG* FISH (21q22) Del, Break, TC (Kreatech Diagnostics, Netherlands) consists of three probes: R, G, and blue (B). A nonrearranged case would show a triplet of RGB signals; however, a translocated case would be represented by one triplet signal corresponding to the normal allele accompanied by another B and G fused and a separated R signal. On the contrary, an interstitial deletion would be shown by one fused triplet signal plus a B and R fused signal. Another predominant pattern consisting of one fused signal plus a B and G adjacent signal was also observed, called the break pattern. Moreover, a fused RGB signal with a separated B/G or R signal alone was assigned to unknown rearrangement (Figure 1).

A total of 50 nuclei of each of the three cores per case were counted. The cutoff was established using the 20 prostate tissue controls by counting 150 cells looking for the translocation. After samples were evaluated, the average and standard deviation of the nuclei with rearrangement were calculated. Then, a cutoff percentage was calculated as 3 positive standard deviations of the average percentage of rearrangement observed in normal tissues. Finally, this percentage was established in 15% of cells showing an altered FISH pattern.

### 2.5. *TMPRSS2-ERG* Detection by RT-PCR

**Figure 2:**
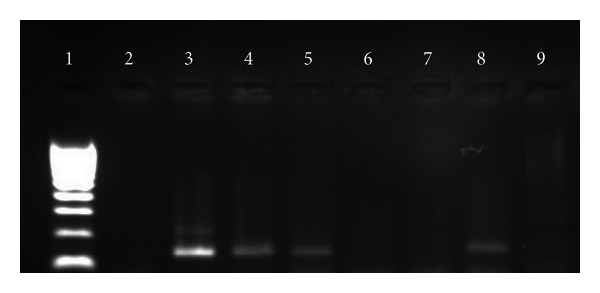
Gel electrophoresis showing representative results for the *TMPRSS2-ERG* gene status determined by RT-PCR. Lane 1: size marker; lines 3, 4, 5, and 8: positive cases showing a PCR product between the bands of 100 and 200 bp of the size marker; lanes 2, 6, and 7 are negative cases, and lane 9 is a negative template control.

One representative FFPE block was identified from each case and three sections of 20 *μ*m thick were obtained for total RNA extraction. The RNA quantification, RT-PCR, and the identification of *TMPRSS2-ERG* fusion transcripts were carried out as previously described in [18] (Figure 2).

PCR products of those *TMPRSS2-ERG* positive cases were purified, quantified, and sequenced on an ABI3130xl sequencer using the BigDye terminator v3.1 kit (Applied Biosystems, Inc., Foster City, CA) with specific primers. Sequencing Analysis v5.2 software (Applied Biosystems, Inc., Foster City, CA) and NCBI blast tool (http://www.ncbi.nlm.nih.gov/BLAST/) were used to confirm the sequences involved in the *TMPRSS2-ERG* fusions.

### 2.6. Performance Test

Considering FISH as gold standard, the sensitivity, specificity, positive predictive values (PPV) and negative predictive values (NPV) were calculated for RT-PCR technique.

### 2.7. Statistical Analysis

Binary variables were used for the statistical analysis reflecting the positivity status of the measures. The association between *TMPRSS2-ERG *and clinicopathological parameters (categorical) was assessed using a chi-square test to determine homogeneity or linear trend for ordinal variables. The significance level was established at 5%. The impact of the biological factors on biochemical (BPFS) and clinical progression free survival (PFS) was analyzed by Log-rank tests. Biochemical progression was defined as serum PSA level >0.4 ng/mL during followup, whereas clinical progression was defined as local (prostatic fossa), regional (lymph nodes), or distant (metastasis). Univariate predictors of both BPFS and PFS were entered into a Cox proportional hazards model using stepwise selection to identify the independent predictors of poor outcome, with a confidence interval (CI) of 95%. Statistical analysis was carried out using the SPSS statistical software package (version 15.0.1, SPSS Inc., Chicago, IL, USA).

## 3. Results

Since the three-color assay is a novel approach for *TMPRSS2-ERG *analysis, a break-apart FISH was first conducted in two TMAs including 38 cases in order to validate the tricolor Kreatech probes. Both approaches presented good concordance measured by chi-square test with a concordant rate of 80.6%, a 13.8% false positive rate (FP), and 15.8% false negative rate (*P* = 0.013) taking break-apart assay as the gold standard.

For the whole series, using tricolor probes, 162 out of the 294 PCa samples (55.1%) were positive for genetic rearrangement. One hundred nineteen of these positive cases (40.5%) showed a break-apart pattern and 35 (11.9%) an interstitial deletion pattern. The remaining 8 (2.7%) cases corresponded to undetermined fusion mechanisms. The presence of *TMPRSS2-ERG *rearrangement or the type of fusion mechanism assessed by means FISH showed no correlation with any of the clinicopathological parameters studied. There was only a trend towards statistical significance between the presence of perineural invasion and the fusion gene detected by the tricolor FISH assay (*P* = 0.054) (Table 2).

With the RT-PCR approach, 152 out of the 294 (51.7%) showed expression of the *TMPRSS2-ERG* fusion gene. One hundred and twenty-nine cases (43.9%) harbored the T1E4 variant (*TMPRSS2 *exon 1 fused to the exon 4 of *ERG*), whereas 11 (3.7%) expressed the T1E5 variant. The identification of fusion gene variant was not possible in two (0.7%) cases.

Taking tricolor FISH as gold standard for determination of *TMPRSS2-ERG*,RT-PCR showed a sensitivity of 79.3% and a specificity of 81.9% with a Positive Predictive Value (PPV) of 80.9% and a Negative Predictive Value (NPV) of 80.2%. FISH and RT-PCR assays showed concordant results in 237 out of 294 cases (80.6%, *P* < 0.001) (Table 3).

Since FISH and RT-PCR showed a good agreement in the assessment of *TMPRSS2-ERG* status, we considered those cases as positive which presented the rearrangement determined by any of the two procedures. The presence of *TMPRSS2-ERG *according to this criterion showed no correlation with any of the clinical and pathological parameters (Table 4). However, and as aforementioned, there was a trend towards the statistical significance between the presence of *TMPRSS2-ERG* and perineural invasion (*P* = 0.091).

**Figure 3:**
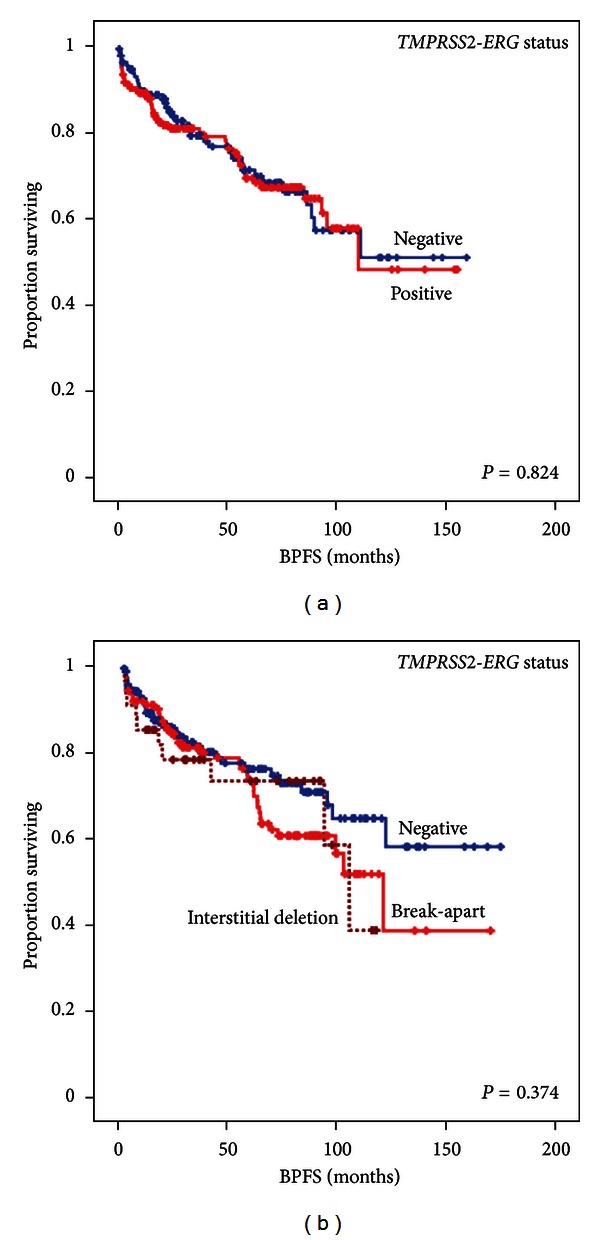
Kaplan-Meier plots of the univariate survival analysis. (a) Biochemical progression free survival (BPFS) according to the *TMPRSS2-ERG *status measured by FISH and/or RT-PCR. (b) BPFS according to the mechanism of the rearrangement determined by tricolor FISH assay.

Regarding the prognostic implications of the presence of gene fusion, Table 5 shows the results of both the univariate and multivariate analyses of the clinicopathological and molecular parameters for both BPFS and PFS. Neither the *TMPRSS2-ERG* status nor the mechanisms of genetic rearrangement were related to the clinical outcome in our series (Figure 3).

## 4. Discussion

Currently, the standard diagnostic method for PCa is the pathological evaluation of prostate biopsy in patients with an elevated serum PSA level and/or an abnormal DRE. However, this clinical approach lacks sufficient sensitivity [19] being necessary for the discovery of new biomarkers that can improve the accuracy of PCa diagnosis. The objective of this study was to assess whether *TMPRSS2-ERG *fusion gene determined by tricolor FISH assay or RT-PCR could be used as part of the diagnostic panel of PCa.

Several strategies can be employed when a FISH experiment is designed. For instance, break-apart probes are widely validated in numerous studies constituting a valuable tool for determination of *TMPRSS2-ERG* in PCa [4, 20–22]. However, this approach has its limitations. A positive result indicates that *ERG *is rearranged, but a second determination of *TMPRSS2* would be necessary to confirm the presence of *TMPRSS2-ERG* because there are other genes that could be rearranged with *ERG* such as *NDRG1 *and *SLC45A3* [23, 24]. With a tricolor strategy one determination is enough to demonstrate the involvement of both genes. Furthermore, although the break-apart FISH has been widely used in investigation, this technique does not have diagnostic approval because noncommercial homemade probes are used. However, the Kreatech Poseidon FISH probes have been developed specifically for their use in the clinical context constituting a valuable ancillary test in the PCa diagnosis.

In the series herein presented, the FISH analysis showed that 55.1% of PCa carried the *TMPRSS2-ERG* fusion gene of which 40.5% showed a split signal pattern and 11.9% evidenced an interstitial deletion. Remarkably, none of the analyzed cases showed the balanced translocation pattern consisting of one triplet signal (RGB) plus a B and G adjacent signals with a separated R signal as indicated by the commercial suppliers. However, the most frequent pattern was a triplet fused signal plus a BG signal (the pattern so-called break). This signal might correspond to a balanced translocation in which the R signal is not visible. However, it is also possible that the break pattern indicates an unknown mechanism of rearrangement between *TMPRSS2* and *ERG*. In eight (2.7%) cases harboring the fusion gene, it was not possible to determine the mechanism by which the rearrangement was produced. Seven of these cases were scored as *TMPRSS2-ERG* positive by RT-PCR, indicating a rearrangement between both genes. The frequency of the *TMPRSS2-ERG* is consistent with the series already published, ranging from 15% to 78% [14, 17, 25, 26]. However, some differences might be explained by variations in the cohorts under study, low number of cases in some series, or sample selection bias [17, 25]. 

When referring to FISH, there are many questions associated with the interpretation of the results, for instance, the presence of multiple signals representing polyploidy or multiple copies of *TMPRSS2-ERG* difficult to interpret [27]; the number of nuclei that should be counted; and, the score of rearranged nuclei to be scored as positive [27, 28]. In our series, we evaluated a median of 150 nuclei per case and selected a 15% of rearranged nuclei as the optimum detection for *TMPRSS2-ERG*, and that discriminates between PCa and normal prostate tissue. This cutoff is similar to the one used by Machado et al. in the determination of *EWSR1* in Ewing sarcoma by means of break-apart assay [27].

Unlike FISH, RT-PCR provides some advantages such as lower cost of the procedure and its capacity of discriminating different variants of the *TMPRSS2-ERG* fusion gene. In this regard, some authors have shown an association between some of these fusion subtypes with good [29] and poor prognoses [30]. However, because of its high sensitivity and cross-contamination, RT-PCR may on occasion give false positive results. Hence, RT-PCR is an interesting and useful technique in the diagnostic setting and should be considered as potential complement to FISH.

To our knowledge, there is only one study comparing FISH and RT-PCR in the determination of *TMPRSS2-ERG *in PCa showing a concordance of 84.7% [21], very similar to the herein reported. In the literature, there are other examples that compare FISH and RT-PCR in different settings [27, 31, 32]: in breast cancer the comparison between both techniques in the determination the *HER2 *status has shown a good concordance ranging between 80–97% [33–35]; in the case of dermatofibrosarcoma protuberans, this concordance is of 67% for detection of *COL1A1-PDGFB *rearrangement [28]; and finally, in Ewing Sarcomas, several studies showed concordances between 55.5 and 100% [31, 36–38].

Regarding the association with clinical and pathological parameters, no correlation between the rearrangement mechanisms and the surrogate prognostic parameters was found. A trend towards statistical significance was observed between the presence of *TMPRSS2-ERG* and a higher perineural invasion, which is a surrogate parameter of poor prognosis, although as an individual variable it loses its importance as a prognostic value in most of multivariate analyses.

One important question to be addressed in this context is the prognostic implications of the presence of fusion gene in PCa samples. To date, it remains controversial, and there are many studies defending the relationship between *TMPRSS2-ERG *and both good [29, 39, 40] and poor [12, 17, 30, 41] prognoses. In addition, other many authors find no correlation between the presence of *TMPRSS2-ERG* and PCa outcome [42]. Interestingly, Attard et al. reported that the presence of *TMPRSS2-ERG* resulted from an interstitial deletion accompanied by a high copy number of this gene (the so called class 2+ Edel), with a poor prognosis [12], but to date, there are no studies confirming these observations. A recent work of Tomlins et al. reported a prognostic panel composed by *TMPRSS2-ERG *and *PCA3* measured in urine samples by means of transcription-mediated amplification [43]. In addition, our group reported the different prognostic panel in PCa that harbors the fusion gene indicating that the determination of this biomarker would be useful in distinguishing groups of PCa patients defined by differential prognostic indicators [18].

Despite the great number of studies published on this matter, and due to differences with regard to the technology used to determine *TMPRSS2-ERG* status and the heterogeneity of the cohorts, it is difficult to draw conclusions about the prognostic involvement of this biomarker. Herein we have analyzed a well-defined cohort with a long followup in order to avoid these problems and no differences between the *TMPRSS2-ERG* status and the genetic mechanisms by which it has been produced with the prognosis of patients with PCa have been found.

In summary, we have demonstrated the usefulness of a commercial tricolor probe FISH approach for the identification for the *TMPRSS2-ERG* status and the genetic mechanism responsible of this fusion gene, as well as high grade of concordance between this strategy and RT-PCR. Furthermore, we have proved that the *TMPRSS2-ERG* status, although specific for PCa, is not valid as a prognostic biomarker in PCa patients treated with radical prostatectomy.

## Figures and Tables

**Table 1:** Demographics and main clinical and pathological features of the analyzed series.

Parameters	*n*	%
PSA		
<10 ng/mL	186	63.3
10–20 ng/mL	66	22.4
>20 ng/mL	42	14.3
Gleason-sp		
≤6	118	40.1
7	139	47.3
8–10	37	12.6
cT		
≤cT2b	273	92.9
≥cT3a	19	6.5
pT		
≤pT2	160	54.4
≥pT3	133	45.2
pN*		
pN0	190	64.6
pN ≥ 1	10	3.4
Perineural invasion		
Negative	126	42.7
Positive	145	49.2

SP: specimen, cT: clinical stage, pT: pathological stage, PSA: prostatic-specific antigen, and pN: pathologic stage with respect to lymph node status.

*Lymphadenectomy was limited to the obturator fossa in most of the cases at the inclusion period.

**Table 2:** Correlation between the mechanism of the rearrangement and the clinical and pathological parameters.

Parameters	*TMPRSS2-ERG* negative (%)	Break apart (%)	Interstitial deletion (%)	*P*
PSA				
<10 ng/mL	85 (47)	73 (73)	23 (23)	
10–20 ng/mL	29 (45.3)	29 (45.3)	6 (9.4)	0.907
>20 ng/mL	18 (43.9)	17 (41.4)	6 (14.6)	
Gleason-sp				
2–6	56 (49.6)	45 (39.8)	12 (10.6)	
7	57 (41.9)	61 (44.9)	18 (13.2)	0.693
>7	19 (51.4)	13 (35.1)	5 (13.5)	
cT				
≤cT2b	122 (45.9)	109 (41)	23 (23)	0.251
≥cT3a	9 (50)	9 (50)	0 (0)	
pT				
≤pT2	75 (48.7)	60 (39)	19 (12.3)	0.639
≥pT3	57 (43.5)	58 (44.3)	16 (12.2)	
pN*				
pN0	84 (45.4)	75 (40.5)	26 (14.1)	0.177
pN ≥1	2 (20)	7 (70)	1 (10)	
Perineural invasion				
Negative	63 (51.2)	44 (35.8)	16 (13)	*0.054 *
Positive	53 (38.1)	70 (50.4)	16 (11.5)	

**Table 3:** Crosstabs with the parameters of the comparison between RT-PCR and FISH techniques.

	Fish		*P*
	Negative	Positive	
RT-PCR			
Negative	122 (80.2%)	27 (19%)	<0.001
Positive	30 (19.7%)	115 (81%)	

**Table 4:** Correlation between the presence of *TMPRSS2-ERG* determined by FISH and/or RT-PCR and the CPP.

Parameters	*TMPRSS2-ERG* Negative (%)	*TMPRSS2-ERG* Positive (%)	*P*
PSA			
<10 ng/mL	85 (64.4)	101 (62.3)	
10–20 ng/mL	28 (21.2)	38 (23.5)	0.899
>20 ng/mL	19 (14.4)	23 (14.2)	
Gleason-sp			
2–6	50 (37.9)	68 (42)	
7	61 (46.2)	78 (48.1)	0.292
>7	21 (15.9)	16 (9.9)	
cT			
≤cT2b	123 (93.2)	150 (93.8)	0.514
≥cT3a	9 (6.8)	10 (6.3)	
pT			
≤pT2	74 (56.1)	86 (53.4)	0.369
≥pT3	58 (43.9)	75 (46.6)	
pN*			
pN0	82 (96.5)	108 (93.9)	0.317
pN ≥ 1	3 (3.5)	7 (6.1)	
Perineural invasion			
Negative	61 (51.7)	65 (42.8)	*0.091 *
Positive	57 (48.3)	87 (53.3)	

*Lymphadenectomy was limited to the obturator fossa in most of the cases.

**Table 5:** Log-rank and Cox regression tests for BPFS and clinical PFS in the 294 cases analyzed.

Parameters	*n*	Biochemical progression						Clinical progression					
		Events	%BPFS	*P*-univariate	HR	95% CI	*P*-multivariate	Events	% Clinical PFS	*P*-univariate	HR	95% CI	*P*-multivariate
PSA													
≤10 ng/mL	186	37	53.6		1		**0.001**	20	77.7				
10–20 ng/mL	66	26	50.4	**<0.001**	2.9	1.6–5.3	**<0.001**	18	52.3	**0.015**	NS		
>20 ng/mL	42	20	28.2		1.6	0.8–3	**0.118**	12	51.9				
Gleason-sp													
2–6	118	21	67.7		1		**<0.001**	11	78.4		1		**0.001**
7	139	39	46.3	**<0.001**	4.4	2.2–8.5	**<0.001**	26	59	**<0.001**	4.8	2–11.1	**<0.001**
>7	37	23	0		3.2	1.8–5.7	**<0.001**	13	48		2.5	1.3–5	**0.008**
cT													
≤cT2b	274	72	51.4	**0.001**	NS			45	65.7	0.410	NS		
≥cT3a	19	11	26.4					5	65.4				
pT													
≤pT2	161	24	68.3	**<0.001**	NS			14	77.8	**0.001**	NS		
≥pT3	133	59	35.4					36	56.7				
pN													
pN0	66	125	50	**<0.001**	1		**0.033**	40	67.6	**0.004**	NS		
pN ≥ 1	9	1	0		2.3	1.1–5.1		5	33.8				
Perineural inv.													
Negative	126	24	56.9	**<0.001**	1		**0.001**	13	82.9	**0.001**	1		**0.013**
Positive	144	58	44.7		2.4	1.4–4		36	50.8		2.3	1.2–4.5	
*TMPRSS2-ERG *													
Negative	132	31	58.7					18	74.3				
Translocation	118	39	39.3	0.229				24	60.1	0.380			
Deletion	35	10	39.4					6	72.4				
Fusion gene status													
Negative	127	35	51	0.824				22	68	0.855			
Positive	166	47	48.2					26	67.7				

SP: specimen, BPFS: biochemical progression free survival, PFS: progression free survival, and NS: not significant.
